# Spike sorting for polytrodes: a divide and conquer approach

**DOI:** 10.3389/fnsys.2014.00006

**Published:** 2014-02-10

**Authors:** Nicholas V. Swindale, Martin A. Spacek

**Affiliations:** Department of Ophthalmology and Visual Sciences, University of British ColumbiaVancouver, BC, Canada

**Keywords:** spike sorting, polytrodes, clustering, tetrodes, multichannel electrodes

## Abstract

In order to determine patterns of neural activity, spike signals recorded by extracellular electrodes have to be clustered (sorted) with the aim of ensuring that each cluster represents all the spikes generated by an individual neuron. Many methods for spike sorting have been proposed but few are easily applicable to recordings from polytrodes which may have 16 or more recording sites. As with tetrodes, these are spaced sufficiently closely that signals from single neurons will usually be recorded on several adjacent sites. Although this offers a better chance of distinguishing neurons with similarly shaped spikes, sorting is difficult in such cases because of the high dimensionality of the space in which the signals must be classified. This report details a method for spike sorting based on a divide and conquer approach. Clusters are initially formed by assigning each event to the channel on which it is largest. Each channel-based cluster is then sub-divided into as many distinct clusters as possible. These are then recombined on the basis of pairwise tests into a final set of clusters. Pairwise tests are also performed to establish how distinct each cluster is from the others. A modified gradient ascent clustering (GAC) algorithm is used to do the clustering. The method can sort spikes with minimal user input in times comparable to real time for recordings lasting up to 45 min. Our results illustrate some of the difficulties inherent in spike sorting, including changes in spike shape over time. We show that some physiologically distinct units may have very similar spike shapes. We show that RMS measures of spike shape similarity are not sensitive enough to discriminate clusters that can otherwise be separated by principal components analysis (PCA). Hence spike sorting based on least-squares matching to templates may be unreliable. Our methods should be applicable to tetrodes and scalable to larger multi-electrode arrays (MEAs).

## Introduction

A classical technique for studying the brain is to record electrical signals with a microelectrode placed near the cell body of a neuron. Action potentials generated by the neuron are detectable as brief (<1 ms) small (<1 mV) changes in electrical potential. These are conventionally referred to as “spikes.” In recent years physiologists have exploited the advantages offered by recording with electrodes that have multiple recording sites close enough to record the same neuron on several adjacent sites. Tetrode electrodes (Reece and O'Keefe, 1989) have 4 recording sites that are typically 25–50 μm apart. Polytrode electrodes (Drake et al., 1988; Bragin et al., 2000; Buzsáki, 2004; Blanche et al., 2005) typically have 1–3 columns of 8–64 channels spaced 50–70 μm apart. Larger multi-electrode arrays (MEAs; Litke et al., 2004; Segev et al., 2004; Frey et al., 2009) designed for recording from retinal patches or brain slices may have hundreds or even thousands of sites. All of these types of electrode are designed so that a given neuron will produce a characteristic pattern of voltage change on a number of adjacent recording sites depending on the position of the unit relative to the sites. This has the advantage that the spike signature of a given neuron can be defined by the voltage change on several different channels, allowing for better discrimination of units (Blanche et al., 2005). However, the overlap, and the large numbers of channels present on most polytrodes and MEAs pose a problem for spike sorting. The dimensionality of the space in which the signals are present (number of channels × number of voltage samples per channel) is large and it is hard to reduce it to a single low dimensional space in which clustering of spike shapes might be done (Einevoll et al., 2012). Additional factors that make sorting difficult are (a) variability in spike shape of single units over time (Fee et al., 1996a,b; Quirk and Wilson, 1999); (b) similarity in spike shapes between neurons; (c) the frequently non-Gaussian nature of the noise in the clusters (Fee et al., 1996a) and (d) the large amount of data (hours of recording and millions of spikes leading to file sizes of several GB) that may have to be processed.

Relatively few of the papers published on spike sorting in recent years propose solutions that address all of the above problems. Many deal with only single or independent channel sorting (e.g. Zouridakis and Tam, 2000; Quiroga et al., 2004) or tetrodes (e.g. Gray et al., 1995; Fee et al., 1996a; Nguyen et al., 2003; Gasthaus et al., 2009; Franke et al., 2010) and do not specifically address the problems caused by spatially overlapping spikes on many channels. Many address specific problems, e.g. non-stationary spike shapes (Bar-Hillel et al., 2006; Wolf and Burdick, 2009; Calabrese and Paninski, 2011) but do not scale well with numbers of spikes, clusters or channels. Many papers assume that clusters are Gaussian in shape (Harris et al., 2000; Nguyen et al., 2003; Litke et al., 2004; Hazan et al., 2006) and/or use clustering methods that are slow and/or require a high degree of user intervention (Meister et al., 1994; Gray et al., 1995; Segev et al., 2004). Recent solutions that have been proposed specifically for retinal MEAs (Segev et al., 2004; Prentice et al., 2011; Jäckel et al., 2012; Marre et al., 2012) all take the approach of identifying a set of spike templates from a limited sample of recording data and then use template matching to identify spikes in the remaining data. This strategy may be appropriate for the retina, where most cells can be expected to fire during the initial sampling period and electrode or tissue drift is not a major problem. However, for cortical recordings, where units fire less predictably, there is a serious risk of missing units which fire at low rates or episodically during the recording period. In addition, these methods are all (reportedly) labor intensive and slow and users often resort to manual determination of cluster boundaries (Einevoll et al., 2012).

In this paper we present a “divide and conquer” approach to sorting spikes recorded with 54 channel polytrodes. It has the aims of being (a) scalable with the number of electrode channels and the number of spikes; (b) fast; (c) substantially automated and (d) complete—i.e. that it addresses all stages of sorting. Following event detection, signals are initially divided into channel-based clusters i.e. one (potentially multi-unit) cluster per electrode channel. Using the principal components derived from the voltage values on the central and immediately neighboring channels, each initial cluster is sub-divided into as many distinct clusters as possible. These are then recombined on the basis of pairwise tests into a final set of clusters. Pairwise tests are also performed to establish the degree to which each cluster is distinct from the others. Because clustering is only ever done in the space defined by the voltages on a central channel and its neighbors, the number of processing steps will scale linearly with the number of channels on the electrode and hence the method should be scalable to larger arrays. This procedure of breaking down and recombining clusters is similar to one proposed earlier by Fee et al. (1996a). Clustering is done with an algorithm, here termed gradient-ascent clustering (GAC) which is based on the mean-shift algorithm of Fukunaga and Hostetler (1975). The procedure is automated except for a final stage where the user reviews cluster pairs and waveform shapes and decides whether clusters are distinct or should be merged.

## Methods

### Data acquisition

The method was developed and tested with signals recorded with 54-site polytrodes (University of Michigan Center for Neural Communication Technology and NeuroNexus: http://www.neuronexustech.com/) placed into the visual cortex of cats anesthetized either with isoflurane and nitrous oxide (Blanche et al., 2005) or with a mixture of propofol and fentanyl. Eye movements were prevented either by continuous infusion of pancuronium bromide or by retrobulbar injections of α-bungarotoxin (Tocris). Following a craniotomy the dura was carefully removed and a nick made in the pia with an ophthalmic slit knife. The electrode was then inserted into the cortex, under visual control, so that the upper recording sites lay just below (~100–200 μm) the visible top surface of the cortex. The plane of the electrode was parallel to the medio-lateral axis with the recording sites facing anterior. The insertion angle was as nearly perpendicular as could be judged to the cortical surface. Following insertion, cerebrospinal fluid (CSF) was wicked away and the craniotomy was filled with an agarose gel (2.5%, Type III-A, Sigma-Aldrich, St. Louis, MO) in artificial CSF at 38–40°C. Experiments on each animal lasted 2–3 days. Single periods of continuous data acquisition lasted typically from 15 to 45 min and consisted of recordings of spontaneous as well as visually driven activity. Visual stimuli included white noise (m-sequence) stimuli, moving bars, sine wave gratings and natural scene stimuli. The signals coming from the recording channels on the electrode were amplified and band pass filtered from 500 to 6 KHz before being digitized with 12 bit resolution at a rate of 25 KHz (a sampling interval of 40 μs). Continuous acquisition of samples at this rate resulted in file sizes of about 8 GB for 45 min of recording.

These and other details of the experimental procedures (Blanche et al., 2005) were carried out in accordance with guidelines established by the Canadian Council for Animal Care and institutional protocols approved by the Animal Care Committee of the University of British Columbia.

Recording sites on the electrode are referred to here as channels. On any particular electrode, usually 2–3 channels were non-functional and connected to ground. They were identifiable on the basis of a low noise level and were masked out from subsequent processing. Electrodes were either of 2 or 3 column design (Blanche et al., 2005) with sites 50, 65, or 75 μm apart.

### Pre-processing

Inspection of Fourier power spectra of raw voltage recordings containing action potentials showed that recorded spikes contributed energy spread across a wide range of frequencies. This suggests that smoothing or other frequency-based (linear) operations on the signal are unlikely to increase the spike-signal-to-noise ratio. We nevertheless tested a variety of pre-processing options, described as follows.

#### Removal of common mean

Subtraction of the common mean when a large number of channels is being used has been shown to be advantageous by Ludwig et al. (2009). The underlying argument is that some sources of noise, especially those from non-physiological external sources, will be common to all channels and this signal can be estimated by taking the mean of all the signals and subtracting it from each channel. This method will work if the internal signals of interest are all uncorrelated across channels and if the number of channels is large. Neither of these assumptions is completely correct but the approximation may be good enough to be useful. We implemented subtraction of the common mean according to the following formula:

(1) $$V^{\prime }\left(n,\;t\right)=V\left(n,\;t\right)-\frac{1}{N_{c}}\sum_{n\;=\;1}^{N_{c}}V(n,\;t)$$

where *V*′(*n*, *t*) is the transformed voltage, *V*, recorded on channel *n* at the integer time point *t*, (in units of 40 μs) and *N*_*c*_ is the total number of channels.

#### Temporal smoothing

A simple and fast way to remove noise is to locally smooth each waveform, such that:

(2) $$V^{\prime }\left(n,\;t\right)=0.5\;V\left(n,\;t\right)+0.25\;V\left(n,\;t+1\right)+0.25\;V\left(n,\;t-1\right).$$

This can be repeated and, with repetition, approximates convolution of the waveform with a Gaussian kernel whose width increases with the number of repetitions. Correspondingly, the Fourier spectrum of the original waveform is multiplied by a Gaussian (whose peak is at zero frequency) thereby implementing a gradual attenuation of high frequencies in the waveform.

#### Spatial smoothing

We sometimes averaged signals across neighboring channels at the same time point. This was done by convolution with a Gaussian with a width comparable to the spacing between adjacent electrode channels:

(3) $$V^{\prime }\left(n,\;t\right)=\sum_{m\;=\;1}^{N_{c}}V\left(m,\;t\right)G\left(n,\;m,\;\sigma_{a}\right)$$

where *G*(*n*, *m*, σ_*a*_) is a Gaussian function of distance, in microns, between electrode sites *n* and *m*, and σ_*a*_ gives the width of the Gaussian. Combining spatial smoothing, e.g. with σ_*a*_ = 50 μm, with temporal smoothing yielded extremely clean-looking signals with almost no noise other than what looked like spikes. However, there was no clear indication that either event detection or sorting were improved as a result.

#### Sample-and-hold correction

Voltage samples from different channels are acquired at slightly different times because the A-D converters (we used two boards with 32 channels each, running in parallel) sample channels sequentially (Blanche and Swindale, 2006) with 1 μs holds (or delays) between consecutively sampled channels. This resulted in sampling delays up to 31 μs across pairs of channels. This is an appreciable fraction of the sampling interval of 40 μs. The required correction was made by recalculating voltage waveform on each channel, interpolating so that voltage values were shifted by amounts that brought the waveform on each channel into exact temporal alignment. Although this correction should probably always be made, we found it made little difference to the results and it was generally omitted.

In summary, pre-processing of the voltage waveforms was either omitted or was limited to removal of the common mean and a single temporal smoothing pass (Equation 2).

### Overview of sorting procedure

**Figure 1:**
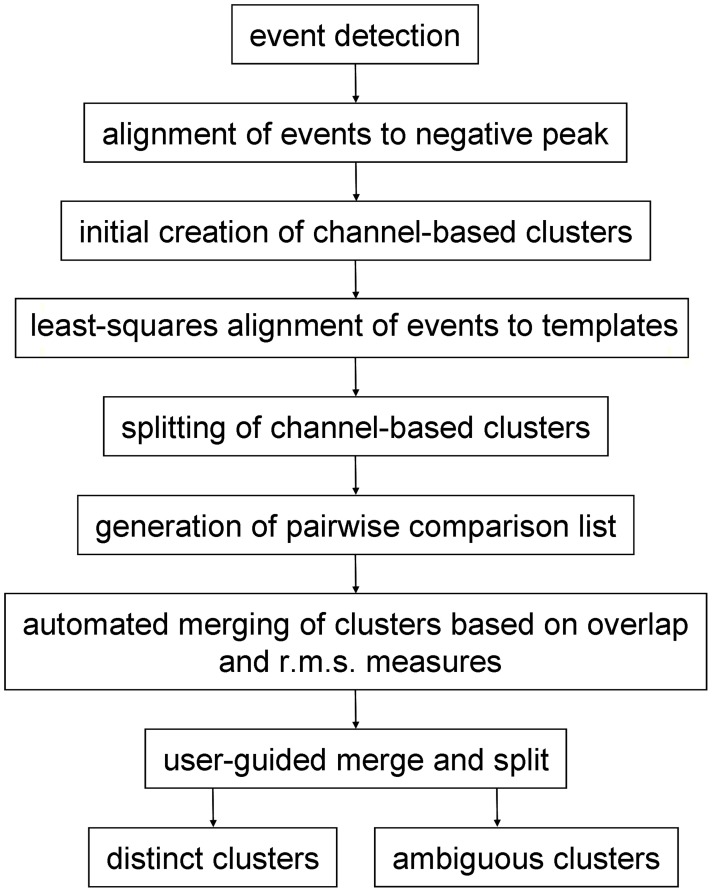
**Stages of the procedure for spike sorting**.

Spike sorting following pre-processing (if any) had the following stages (Figure 1): (a) event detection followed by initial event alignment; (b) assignment of events to channels to form an initial set of channel-based clusters and then (c) splitting (or sub-clustering) these events into single, homogenous clusters. Because single units may give rise to events that are inconsistently assigned to different (usually neighboring) channels, and therefore end up in different clusters, a further stage was needed, (d) where clusters were either merged or events were reassigned between pairs of neighboring clusters. Clusters that were objectively distinct from all other clusters were identified during this stage. In the final stage (e) the user reviewed cluster pairs that could not be defined as distinct by automated procedures. Pairs could be merged, recombined and split, or declared to be distinct by the user. Ambiguous clusters—those which failed the distinctness test for one or more cluster pairs—could either be deleted or treated as incomplete and/or multi-unit.

### Event detection

The event detection method we used will not be described in detail here on the grounds that it is conceptually a separate stage of spike sorting. We emphasize that the sorting methods we describe can be applied to events detected by any method. We will however, give a brief justification and description of the event detection method we used, as follows.

A problem specific to event detection on multichannel electrodes is that a single spike may give rise to detectable events on several adjacent channels. Existing methods (e.g. see Blanche, 2005) consist of applying a threshold test of some kind to the waveform on a particular channel, *n*, at some time *t*, and registering an event if the waveform passes a further test (e.g. falls above or below a second threshold, or thresholds, at one or more points in time following *t*). Following event registration at (*n*, *t*) a spatiotemporal lockout is applied, meaning that events are vetoed from detection for a short time (typically *t* ± 0.5 ms) on the same channel. Events detected at the same time on channels some specified distance away (typically <150 μm) are also locked out on the grounds that they are likely to be caused by the same spike. Spatiotemporal lockout is problematic however given that spikes from different neurons may occur close together in time and space. Whichever of the two events is detected first will cause the other one to be locked out even though the two events might otherwise be identifiable as two. This is undesirable because as well as resulting in undetected events, measures of spike synchrony between nearby cells at short time intervals will yield fewer synchronous or near-synchronous events than might actually be occurring. The fact that a single real event (i.e. a spike) may give rise to multiple detected events on adjacent channels suggests that event detection on polytrodes is, for most spikes, a clustering problem. Events detected at nearby positions in space and time may be caused by a single spike, though sometimes adjacent, or nearly adjacent, events may be caused by different spikes. The way in which the events are clustered in space and time however, may give clues as to whether they are caused by single or multiple spikes.

**Figure 2:**
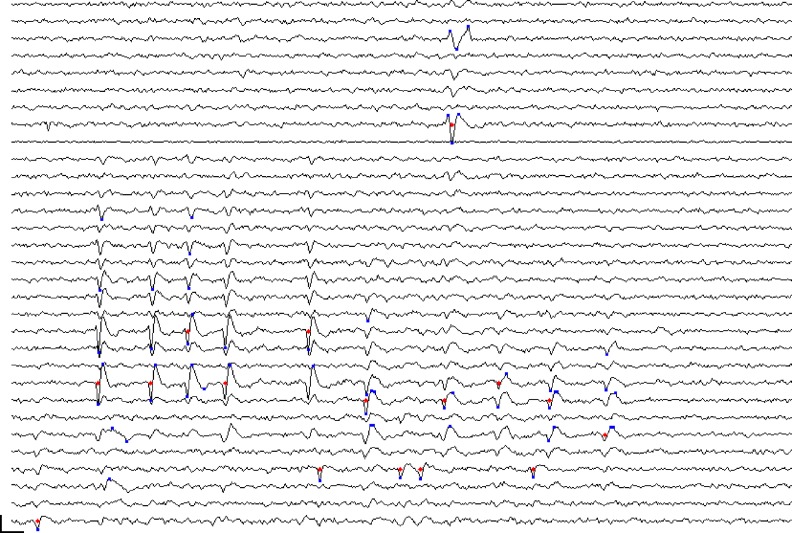
**Event detection: section of recorded voltage waveform showing proto-events—local maxima or minima in space-time (blue dots)—and the resulting event locations (red dots) on the assigned event channel (*n*_*i*_) following proto-event clustering**. Recording channels are arranged in order of vertical position on the polytrode, which had three columns. Spatially adjacent channels are thus not always adjacent on the figure. One flat channel (9th from the top) is masked out from processing. Note the inconsistent assignment of events that are probably from the same unit to different channels. The two waveforms marked by proto-events at the top (3rd and 8th channels from the top) are on channels that are close together and were recognized as a single event. Subsequent clustering confirmed that these were not two separate units. Scale bar = 1 ms and 100 μV.

Given this, we accomplished event detection by first registering points in time and space, referred to here as proto-events, which passed a threshold test. We defined proto-events as thresholded, local voltage space-time maxima or minima. That is, a point (*n*, *t*) was registered as a proto-event if (a) its voltage *V*(*n*, *t*) exceeded the voltage in all the nearest neighbors of channel *n* in the array at time *t*, and if it exceeded the values of *V*(*n*, *t* −1) and *V*(*n*, *t* +1), and (b) if *V*(*n*, *t*) exceeded a threshold voltage. The inverse of this was also applied, i.e. a point was registered as a proto-event if *V*(*n*, *t*) was a local minimum and if *V*(*n*, *t*) fell below the same (negative) voltage threshold. Following Quiroga et al. (2004) we defined the voltage threshold separately for each channel as a multiple, θ_*e*_, of the channel noise, measured as the median of the absolute voltage values divided by 0.6745. A single spike might sometimes give rise to only one proto-event if only a single peak crossed threshold; however most spikes gave rise to several proto-events on neighboring channels at points in time corresponding to the peaks and troughs of the waveform. Peak or trough voltages of a spike did not always occur at the same time on adjacent channels and such occurrences likewise could give rise to multiple proto-events. Two spikes that are adjacent in space-time will give rise to clusters of proto-events that might abut but should be recognized as separate clusters. Figure 2 shows a sample portion of recorded waveforms with detected proto-events shown as blue dots and the resulting event cluster centers shown as red dots. We typically used thresholds θ_*e*_, in the range 5.0–6.0 for proto-event detection.

Proto-events were then clustered with the aim that those belonging to the same spike were merged into a single event while those belonging to different spikes were not. The clustering methodology was based on the gradient ascent method described below. Tests and comparisons of the method with more standard event detection methods will be reported in more detail in a separate paper (Swindale and Spacek, in preparation). We emphasize that none of the results presented in this paper depend critically on the use of this particular method and that similar results would be obtained with more standard event detection methods.

Event detection yielded event times, given by the integer *t*_*i*_, where *i* indexes the event, and *t* is the time of the event in multiples of the sampling interval of 40 μs. A channel number, *n*_*i*_, was defined as the channel that was closest to the final position of the event.

### Calculation of templates and event alignment

A number of procedures were common to all stages of sorting. Following assignment of events to a cluster, the average waveform of the events in the cluster, which is here termed the template, was calculated. Prior to, or following, calculation of a template, events may be aligned in various ways. By alignment we mean the choice of an exact time at which the event can be said to have occurred. Alignment may be based on a variety of criteria: (1) *event-based alignment* in which each event is independently aligned based on the shape of the event itself (e.g. to a peak or trough) without reference to a template or other events; (2) *least-squares matching to the template* in which events are aligned to minimize a least-squares match to the template; and (3) *template-based alignment* in which the alignment of all the events in a single cluster is changed by the same amount causing the position of the template to shift.

Alignment is important as it allows more exact comparisons of events and templates for the purposes of splitting and merging clusters. In order to do this we found it necessary to increase the accuracy of representation of event time by calculating a fractional floating point offset, δ_*i*_ based on sinc interpolation of the event waveform between sample points (Pouzat et al., 2002; Blanche and Swindale, 2006). Sinc interpolation was done to 4 μs accuracy (9 interpolated points per sample point, summed over ± 6 sample points). The extra accuracy in alignment of waveforms given by interpolation was found to give a significant advantage in subsequent clustering. In particular it avoided the artifactual splitting of clusters of large-amplitude spikes into two clusters. Storing event times as an integer plus a separate floating point offset also avoided the loss of accuracy that might result from using a single floating point value to store both the integer and fractional parts of the time index since the integer parts could be large (~2^27^) for long (~60 min) recording periods.

The procedures for calculating templates and for aligning events are described in more detail in the following sections.

#### Event-based alignment

Event-based alignment was used immediately following event detection when clusters were not yet present. Options explored included: (a) alignment to the largest peak of the waveform; (b) alignment to the most negative trough; (c) alignment to a measure of the center of the entire waveform or (d) alignment to the position of the first positive-going zero-crossing of the waveform. For the results presented in this paper we chose option (b) since a single negative minimum was often prominent.

#### Calculation of templates

Following the assignment of events to a cluster, or the addition or removal of events from an existing cluster, a template, *T*_*k*_(*n*, τ)—the average waveform of all the events in the cluster—was calculated as:

(4) $$T_{k}\left(n,\;\tau \right)=\frac{1}{N_{k}}\sum_{i\in Q(k)}V^{*}\left(n,\;t_{i}+\delta_{i}+\tau \right)$$

where *k* denotes the template or cluster number, *n* is the channel number, τ is the time index relative to the template center (τ = 0) in integer units of 40 μs, *Q*(*k*) is the set of events, *i*, in cluster *k*, *N*_*k*_ is the number of events in the cluster, *t*_*i*_ + δ_*i*_ is the time (integer plus fractional offset) of event *i*, and V^*^(*n*, *t*) returns the interpolated value of *V* for the non-integer time *t* = *t*_*i*_ + δ_*i*_ + τ. Voltages were calculated for values of τ in the range −10 to 15 (−0.4 to +0.6 ms) inclusive, giving 26 voltage samples per template waveform. To speed up calculations for clusters larger than 1000 events in size, only 1000 randomly chosen events were used to calculate the template. The standard deviation of the voltage values was also calculated.

Following calculation of the template, a set of channels, *P*_*k*_, was assigned to it. A center channel, *n*_*k*_, on which the peak-to-peak voltage was greatest was always assigned. Additional channels were included in the set if the peak-to-peak voltage, *V*_*pp*_ ≥ 0.2 times the peak-to-peak voltage on the center channel, and if the peak-to-peak height on the channel was >2 times the standard deviation of the template measured on its center channel. This typically resulted in the assignment of 3–8 channels per template. A spatial center position, (*x*_*k*_, *y*_*k*_) of the template was calculated as the mean position of the channels in set *P*_*k*_, weighted by the peak-to-peak voltages.

#### Least-squares alignment of events to templates

After the initial calculation of the template, individual events were realigned to it using a least-squares match. This was done by calculating the temporal offset, τ_min_, which when added to the event time, minimized the RMS difference between the event waveform and the template. This is given by

(5) $$\tau_{\min}=\underset{\tau_{s}}{}\arg\min\left\{\sum_{n\subset P_{k}}\sum_{\tau =-10}^{\tau =15}{\left[V^{*}\left(n,\;t_{i}+\tau +\tau_{s}\right)-T^{k}\left(n,\;\tau \right)\right]}^{2}\right\}$$

This was done over a range of values of τ_*s*_ = −5.0 to +5.0 (equivalently ± 200 μs). The event time was then recomputed as *t*′_*i*_ = int(*t*_*i*_ + τ_min_) and δ′_*i*_ = *t*_*i*_ + τ_min_ − *t*′_*i*_.

Equation 5 does not necessarily have a single local minimum, however in nearly all cases (>99.75%) a minimum was found within τ = ± 1. Values of τ_min_ at the extremes of the search range (i.e. = ± 5) generally indicated noisy or spuriously shaped events and these were either left where they were, or removed from the cluster.

Since least-squares realignments changed the shape of the template, it was recalculated following the realignment of events. Further realignments resulted in increasingly small and insignificant changes in template shape and individual event alignment times.

#### Template-based alignment

**Figure 3:**
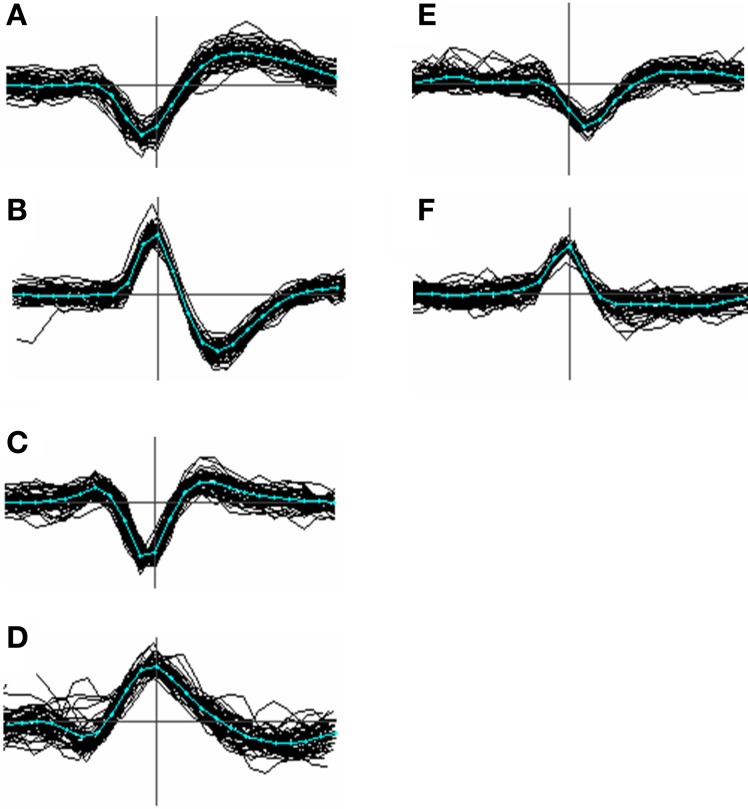
**Menagerie of spike shapes classified according to the presence and temporal order of peaks and troughs**. Types **(A–C)** are the most common; type **(D)** less so; types **(E)** and **(F)** were the closest approach to monopoles we could find in our data and are uncommon (<1%). In order the classifications can be labeled as [−, +], [+, −], [+, −, +], [−, +, −], [−], and [+]. Although these labels can be derived unambiguously from most averaged spike templates, the categories are not clearly distinct, and individual waveforms even less so.

For the purposes of deciding whether template pairs are similar (indicating that they should be merged) or different, it is desirable to align them in a way that makes recognition of similarity easy. Templates can be aligned to the same types of feature used for event-based alignment, such as peaks or zero-crossings. Aligning a template implies realigning all the events within it by adding or subtracting a constant time value to all of the individual event times. This is best done by choosing an alignment criterion that does not depend on a non-linear selection of a feature such as a peak or zero-crossing. For example some spike waveforms may have two troughs of nearly equal amplitude on either side of a larger peak (e.g. see Figure 3D). This can result in templates aligned to different features of what is actually the same event waveform, with the result that they are spuriously assigned to apparently distinct clusters. We chose instead to align templates to a 2nd derivative weighted measure of the average position in time, τ_*k*_, of template *k*:

(6) $${\bar{\tau }}_{k}=\frac{\sum_{n\subset P_{k}}\sum_{\tau =-\tau_{0}}^{\tau_{0}}\tau \left|\frac{d^{2}T_{k}(n,\;\tau )}{d\tau^{2}}\right|}{\sum_{n\subset P_{k}}\sum_{\tau =-\tau_{0}}^{\tau_{0}}\left|\frac{d^{2}T_{k}(n,\;\tau )}{d\tau^{2}}\right|}$$

where $\frac{d^{2}T(n,\;\tau )}{d\tau^{2}}=2T(n,\;\tau )-T(n,\;\tau -1)-T(n,\;\tau +1)$. A relatively long temporal window, with τ_0_ typically = 38 (i.e. 1.5 ms), was used to determine the average position because templates can be extended in time. Ideally, small shifts in the template position should shift τ_*k*_ by the same amount and this will only happen if the entire non-zero portion of the template lies within the range of values of τ_0_. Following calculation of τ_*k*_, the new integer time of each event was defined as *t*′_*i*_ = int(*t*_*i*_ + δ_*i*_ + τ_*k*_) and the new fractional offset was given by δ′_*i*_ = *t*_*i*_ + δ_*i*_ + τ_*k*_ − *t*′_*i*_.

This method had the advantage of being parametric, i.e. small changes in template shape produce only small changes in the template position, unlike non-linear measures based on feature selection. The use of the 2nd derivative weighting tended to bias the center of the template toward the sharpest of the peaks or troughs and to reduce the influence of slow changes following the spike, which could often be quite prolonged.

### Formation of channel-based clusters

Following event detection and initial event-based alignment, an initial set of clusters was formed, one for each non-masked (section Data Acquisition) electrode channel, by assigning all the events registered to a particular channel to the same cluster. The template of each channel-based cluster was then calculated (section Calculation of Templates) and events were realigned to the templates using least-squares matching (section Least-Squares Alignment of Events to Templates).

### Sub-clustering of channel-based clusters

We next carried out a test for the presence of sub-clusters in each of the channel-based clusters. If the cluster was considered to be homogenous and unsplittable it was labeled as such and the algorithm proceeded to the next cluster in the list. Otherwise, the cluster was split according to user defined preferences into two or more sub-clusters. Each of these clusters was then subjected to the same test and split if necessary, until all of the sub-clusters formed from the initial one were judged to be unsplittable. This procedure was repeated for the next channel-based cluster and so on until all the clusters in the list were judged to be individually unsplittable. The sub-clustering was done as follows.

### Extraction of principal components

For each of the *N*_*k*_ multichannel waveforms in the cluster, a data vector was constructed by taking the voltage values at *M* selected time points of the waveform on the channels assigned to the cluster template. We selected the time points by ranking the variances of the *N*_*k*_ voltages at each time point and taking up to *M* = 100 points with the highest variance (the number could be less for channels with few neighbors). From these *N* vectors an *M* × *M* covariance matrix was calculated and the principal component eigenvectors were calculated using standard techniques (Press et al., 1994). These were sorted in order of eigenvalue and the dot products of the first few with each data vector were used as inputs for the subsequent clustering stage. We typically used only the first two or three principal components for clustering.

### GAC clustering based on principal components

The GAC algorithm works in the following way. Data points (in this case the principal component values extracted from the spike waveform) **v**_*i*_ = (*x*_1, *i*_, *x*_2, *i*…_), *i* = 1… *N*, where *N* is the total number of events being clustered, were duplicated to form a second set, **s**_*k*_ = **v**_*i*_, *k* = 1 … *K*; *K* = *N* initially. The points **s**_*k*_ will be referred to as “scout points.” A set of cluster indices, *c*_*i*_ was assigned such that *c*_*i*_ = *i* initially. At each step, each scout point used a Gaussian kernel estimator to calculate a local density gradient from points **v**_*i*_ and moved up the gradient by an amount:

(7) $$\Delta s_{k}=\frac{\sum_{i=1}^{N}\left(v_{i}-s_{k}\right)e^{-\frac{{\left|v_{i}-s_{k}\right|}^{2}}{2\sigma_{m}^{2}}}}{\sum_{i=1}^{N}e^{-\frac{{\left|v_{i}-s_{k}\right|}^{2}}{2\sigma_{m}^{2}}}}$$

This procedure for ascending density gradients is elsewhere termed the mean-shift algorithm (Fukunaga and Hostetler, 1975). Following this step, pairs of points in **s** that came within a distance, ε, of each other were merged, together with their associated cluster indices. This was done by deleting the point with the higher index, then setting those cluster index values that equaled the index of the deleted point equal to the lower index. The values of indices higher than that of the merged point, and the value of *K*, were then decremented by 1. This ensured that cluster indices remained in the range 1 to *K*.

Equation 7 was then recomputed for the remaining scout points and the process of movement followed by merging was repeated until all the points in **s** satisfied a criterion for being stationary. This criterion was that the point should have moved a distance Δ**s** < 0.001 for 25 successive iterations. The end result was a set of *K* clusters with the cluster membership of the *i*-th data point **v**_*i*_ given by the value of *c*_*i*_.

Equation 7 is slow to compute (of order *N*^2^) if the summation is done over all the data points in the cluster. If the number of data points is large, not all of them need to be included in the summation, at the expense of possibly losing some very small clusters. We normally summed over every *m*-th point where *m* = int(*N*/5000) + 1.

The algorithm can be visualized working on a hilly density landscape as follows: at the beginning, each scout point in **s** is labeled with an integer that uniquely identifies the data point in **v** from which it originates. Scouts move uphill and if two meet, one hands over its label, or set of labels, to the other and is deleted. Eventually there remains a single scout at the top of each hill with a set of labels that identifies all the data points that belong to the same cluster. Thus, points that have moved up gradient paths that merge in a common center are considered to be members of the same cluster. The detail, or smoothness, of the density landscape is determined by the value of σ_*m*_. If the data points form well defined, separate clusters there should be a range of values of σ_*m*_ that leads to similar numbers and sizes of clusters.

Stability of cluster sizes was measured by running the algorithm with a series of increasing values of σ_*m*_, referred to below as a clustering pass. The end result of each run, indexed by *m*, is a set of *K*(*m*) sub-clusters, indexed by *k*, with varying numbers of members, *C*^*k*^_*m*_, in each. The value of σ_*m*_ was increased until the number of clusters *K* = 1. To identify stable ranges of σ_*m*_ for particular sub-clusters, they had to be tracked across consecutive values of σ_*m*_. A sub-cluster was identified as the same from one value of σ_*m*_ to the next, if (a) its size changed by less than a criterion percentage θ_*N*_ (typically 5%) and (b) if its position (the mean value of all its members) changed by less than 0.14σ_*m*_. Each sub-cluster was then assigned a stability score, *S*^*k*^_*m*_, which was equal to the number of steps of σ_*m*_ across which it had been tracked. Splitting of the cluster was then determined by the values of *S*^*k*^_*m*_ for the different sub-clusters. If no scores fell above a threshold, θ_*c*_, the cluster was deemed to be unsplittable. We explored the options of (a) choosing to split the single sub-cluster with the best score; (b) choosing the value of σ_*m*_ for which the score summed across all the potential sub-clusters was a maximum; or (c) choosing the value leading to the splitting of the maximum number of clusters. A minimum cluster size, *N*_min_, was also applied during this procedure. Once a best value for σ_*m*_ had been chosen, events in sub-clusters for which *N*_*k*_ < *N*_min_ were deleted, by setting their cluster index to zero.

Specific parameters used for the results reported here were σ_1_ = 5 μV, with σ_*m*_ increasing by 10% on successive iterations and terminating with a value for which *K* = 1; the merge distance ε = σ_*m*_; the % change threshold θ_*N*_ = 5%; the clustering score threshold θ_*c*_ = 8 and the minimum cluster size *N*_min_ = 50. For the results presented in this paper we chose option (a) above, i.e. splitting off the sub-cluster with the highest value of *S*, since (as shown below in section Single Clustering Pass) this often led to an increase in the clusterability of the remaining points.

Whenever a new cluster was formed by the above procedures, or events were removed from a cluster, the template was recalculated and the new or remaining events were aligned to it by least-squares matching. Once a cluster was deemed to be stable, the template was aligned as described above in section Template-Based Alignment in preparation for the following merging and reassignment stage.

### Merging and reassignment of events between clusters

The result of the first clustering stage is the formation of a number of clusters (100–150 is typical) which are individually deemed to be unsplittable. There remains however, the problem that events belonging to a single unit may have been split between adjacent channels. This happens especially for units whose spikes are smaller and have a wider spatial spread than others, or in cases where a more narrowly distributed spike happens to be positioned midway between channels. The splitting may have two possible outcomes. One is that the events end up in two (or sometimes more) clusters which have to be recognized as containing the same class of events and simply have to be merged into one. This outcome is more likely when the two relevant clusters are roughly equal in size. Another occurrence is that a small number of spikes from a cluster get registered to a neighboring channel and end up being included in a larger cluster. Frequently, the same thing happens to spikes in the other cluster. This case requires reassigning spikes between the two clusters which can be done by merging and re-clustering. Another problem requiring merging is that clusters may have been wrongly split because of inconsistent alignment to variably placed negative troughs.

Testing for these cases required comparisons of all pairs of clusters. Given a measure of distinctness between pairs, a cluster was formally defined as “distinct” if it was unambiguously separable from all other clusters. If it was not, then the status was defined as “ambiguous”: spikes might be missing and/or the cluster might contain subsets of spikes from a variety of units with similar shapes. Note that the term “distinct,” if applied to a cluster pair, indicates only that the pair is distinct. Calling a cluster “distinct” implies that it is distinct from all other clusters.

The goal of the second stage of clustering was therefore to apply a distinctness measure to all pairs of clusters, merging clusters and reassigning events with the goal of maximizing the number of distinct clusters. Although the number of pairs is large (for 100 clusters it is 4950) the great majority can safely be declared as distinct because they are physically far apart and have few or no channels in common. We decided to partially automate the procedure, leaving a final set of pairs for which the user was able to decide on the basis of visual inspection whether to merge, merge and re-split, define as distinct or leave as ambiguously related. Two measures of cluster similarity were used as a guide to this process: an RMS measure of template shape similarity and a measure of overlap of the points in clusters pairs in their common principal components space. Template pairs were excluded from this comparison (i.e. were deemed not to overlap spatially) if less than half of the members of both sets of channels assigned to the templates were members of the other set. For example, a template with only two channels will overlap another one if one of the two channels is also assigned to the other template, no matter how many other channels the template has, whereas a template with three channels would not overlap if only one of the three was in common.

#### RMS template similarity

An obvious measure of similarity between two clusters is the similarity between their templates. A measure of this is the RMS voltage difference between the pair, which we calculated as:

(8) $$q_{k,l}={\left\{\frac{1}{M}\sum_{n\subset U_{k,l}}\sum_{\tau =-10}^{\tau =15}{\left[T_{k}\left(n,\;\tau \right)-T_{l}\left(n,\;\tau \right)\right]}^{2}\right\}}^{0.5}$$

where *T*_*k*_(*n*, τ) is the voltage on channel *n*, at time τ of the *k*-th template; *U*_*k*,*l*_ denotes the union of the channels in sets *P*_*k*_ and *P*_*l*_, and *M* is the number of points in the summation (= 26 × number of channels in *U*_*k*,*l*_). The measure is symmetrical (i.e. *q*_*k*,*l*_ = *q*_*l*,*k*_).

#### Cluster pair overlap

Because our primary clustering measure is based on a projection of spike waveform values into a reduced principal components space it seemed appropriate to use the same kind of projection as a measure of overlap between cluster pairs. To do this we temporarily merged the pair, calculated the template, and realigned all the events to it. We then calculated the principal components and projected the points, labeled as to their cluster origin, into the first two dimensions of the space. We refer to plots of points in this common space as cPC plots. If the points have similar, overlapping distributions it should be safe to merge the clusters. If the points form clearly separable clusters then the source clusters can safely be labeled as distinct. For intermediate cases of partial overlap the GAC algorithm can be used to decide if two distinct clusters are present. To assess the distinctness of the distributions, we used an overlap measure defined on the basis of nearest-neighbor identity. For the smaller of the two clusters, labeled *k* and *l*, where *N*_*k*_ < *N*_*l*_ we calculated the number of nearest neighbor points, *u*_*k*_, that belonged to cluster *k*. The proportion, *p*_*k*_ = *u*_*k*_/*N*_*k*_, was compared with the value expected for completely mixed clusters *e*_*k*,*l*_ = *N*_*k*_/(*N*_*k*_ + *N*_*l*_) to give a normalized measure

(9) $$o_{k,l}=\frac{1-p_{k}}{1-e_{k,l}}$$

This measure is close to 1 (it can be slightly greater) for clusters that overlap nearly completely and has a minimum value of 0 for clusters with no overlap, i.e. where all nearest neighbor pairs belong to the same cluster. Calculation of *o*_*k*,*l*_ was sped up by sub-sampling equal fractions from the two clusters so that the total number of points in the comparison was ≤2000. A further increase in speed up came from only calculating it for templates that were deemed to overlap spatially (see above in section Merging and Reassignment of Events Between Clusters).

#### Pairwise distinctness tests

The overlap index could sometimes be low (e.g. <0.1) for cluster pairs whose template shapes, measured by *q*, were similar (e.g. <10 μV). This meant that low values of *q* could not safely be used as a criterion for merging. Conversely, template shapes could be different for clusters pairs whose points were not clearly separated in cPC plots. For these reasons (and others, discussed below in section Merging and Splitting Clusters) we approached the task of automated merging and splitting cautiously, relying largely on the overlap index.

We began by calculating lists of *q*_*k*,*l*_ and *o*_*k*,*l*_ for every pair of clusters. Each cluster, *k*, was then defined as “distinct” if one or more of the following pairwise criteria were satisfied for all other clusters, *l* ≠ *k*:

1. Less than half the channels in set *P*_*k*_ were members of set *P*_*l*_ and *vice versa*;
2. *q*_*k*,*l*_ >25 μV (a conservative criterion that separated clusters with very different waveforms);
3. *o*_*k*,*l*_ < 0.05 (a conservative criterion that identified cluster pairs with very little overlap that could safely be assumed to be distinct with few wrongly assigned spikes);
4. The user indicated that the pair is distinct;

We will refer to this distinctness test as *DT*(*k,l*) returning the value *true* or *false* for a given cluster pair. If a cluster pair fails this test, both clusters are defined as “ambiguous” (= not distinct) no matter what their relations are with other clusters.

In the initial, automated, stage of the merging and reassignment procedure, ambiguous cluster pairs were merged if *q*_*k*,*l*_ < 5.0 μV and if *o*_*k*,*l*_ > 0.9. Following this, pairs for which 0.05 ≤ *o*_*k*,*l*_ < 0.15 (indicating a minor degree of overlap), were then combined and tested for the presence of stable sub-clusters. If these were present (i.e. if the clustering stability score *S* exceeded the threshold θ_*c*_ for one or more sub-clusters) the cluster was split. Since the cluster was the sum of two source clusters, splitting consisted of reassigning points between the original two clusters plus occasional rejection of outlying points that fell into neither sub-cluster. If, on the other hand, the combined cluster was unclusterable, i.e. no clustering score, *S*, exceeded the threshold θ_*c*_, the original two clusters were restored and the relation between them remained ambiguous.

Following each merge or reassignment, the lists of values of *q*_*k*,*l*_ and *o*_*k*,*l*_ were updated by recalculating all of the pairwise measures involving either of the two clusters.

During automated merging and reassignment the number of distinct clusters generally increased. At the end of it, the user was able to serially review a list of cluster pairs that still failed test *DT*(*k,l*). Review involved inspecting the projection of combined pairs into the common (cPC) space together with superimposed plots of spike waveforms from the two clusters. The user could decide to merge two clusters if waveforms appeared similar and if points in the cPC plots appeared to combine to form a homogenous distribution around a common center. Occasionally, waveforms appeared different and yet the cPC plots and overlap measure did not clearly indicate separable clusters. In such cases the user was able to apply rule 4 of test *DT* to indicate that the clusters were distinct. In many cases the cPC plots showed irregular and/or overlapping distributions of points and we generally let such pairs remain ambiguous. Plots showing the times at which pairs of units fired (*x*-axis) together with the peak-to-peak height (*y*-axis) colored according to cluster origin (similar to Figure 10G), as well as cross- and auto-correlograms, were often helpful in deciding whether to combine clusters or not. For example if one unit started firing immediately after the other one stopped, and the peak-to-peak heights were similar at the transition point it seemed likely that they should be combined.

### Code

Program code was written using Intel Visual Fortran Composer XE running under Windows 7 (64 bit) on an Intel Sandy Bridge Core i7 CPU at 3.4 GHz. The entire voltage record (typically 2–8 GB) was read from disk and stored in RAM. A stand-alone Windows program that executes the procedures described in the paper is available on request. Source code is also available if requested. An alternate implementation (http://spyke.github.io) was written in Python and tested in Linux, using free libraries and multithreaded Cython code for computationally expensive steps such as GAC. This implementation works across multiple voltage records of greater total duration, loading data as needed on the fly.

Table 1 lists the more common mathematical symbols used in the above description.

**Table 1:** **Definitions of commonly used mathematical symbols, typical values, and units**.

**Symbol**	**Meaning**	**Range or typical value**	**Units**
**GENERAL**			
*i*	Event number	1–500,000	
*n*	Channel number	1–54	
*N*_*c*_	Number of non-masked electrode channels	51–52	
*V*(*n*,*t*)	Voltage on electrode channel *n* at time *t*	−250 to 250	μV
*t*_*i*_	Integer time index of event *i*	1–10^8^	40 μs
*x, y*	Position in electrode coordinates	0–2500	μs
*Z*_*n*_	The set of immediate neighbors of channel *n*		
δ_*i*_	Fractional offset of the event time relative to *t*_*i*_	0–1	40 μs
θ_*e*_	Threshold for proto-event detection	2–6	
**TEMPLATES**			
*d*_*k*,*l*_	Physical distance between centers of templates *k* and *l*	0–1200	μm
*n*_*k*_	Center channel for template *k*	1–54	
*P*_*k*_	The set of channels assigned to template *k*		
*q*_*k*, *l*_	RMS voltage difference between templates *k* and *l*	3–40	μV
τ	Time offset from template or event center	−10 to +15 (−0.4 to +0.6 ms)	40 μs
*T*_*k*_(*n*,τ)	The template voltage on channel *n*, at time τ, obtained as the mean of the waveforms in cluster *k*	±250	μV
*U*_*k*,*l*_	The union set of channels in templates *k* and *l*		
**CLUSTERING**			
*c*_*i*_	Cluster index assigned to point *i* (of *N* waveforms)	1–1000	
*C*^*k*^_*m*_	The number of points in sub-cluster *k* obtained with σ_*m*_		
*K*	Number of clusters	50–200	
*N*_*k*_	No of points (events) in cluster *k*	50–100,000	
*N*_min_	Minimum cluster size	5–50	
*m*	Clustering step	1–30	
*o*_*k*,*l*_	Overlap between clusters *k* and *l* in cPC space	0–1	
*Q*(*k*)	The set of events *i* in cluster *k*		
*S*^*k*^_*m*_	The clustering score for sub-cluster *k* at σ_*m*_	1–20	
**s**	List of coordinates of scout points		
**v**	List of principal component values for each waveform		
σ_*m*_	Spatial scale for clustering of principal components	5–100	μV
θ_*c*_	Threshold applied to the clustering score, *S*	8	
θ_*N*_	Cluster size change threshold	5	%

## Results

### Template shapes

Figure 3 shows a variety of waveform shapes that we encountered in our recordings, defined in terms of the temporal order and number of peaks and troughs. No claim is made as to the actual distinctness of these types but the variation they embody appears to go beyond that predicted by biophysical modeling studies (e.g. see Gold et al., 2006; Mechler et al., 2011) and is relevant to the problem of alignment. Of the 6 types, type A was the most common, accounting for roughly 80% of the templates. Type B occurred at a rate 1–5%, type C around 15% while types D, E, F were relatively uncommon, accounting for at most 1% each. Types E and F are arguably just extreme cases of type C and D. Types A and B can be aligned consistently based on the choice of either the peak or trough, because (by definition) these types contain only a single peak or trough. Type C events however cannot be consistently aligned based on the use of the maximum peak because, for many of them, there may be two peaks of nearly equal amplitude where one is not consistently larger than the other. This will result in spikes being split into two clusters. A similar argument applies to the troughs of type D spikes.

### Single clustering pass

**Figure 4:**
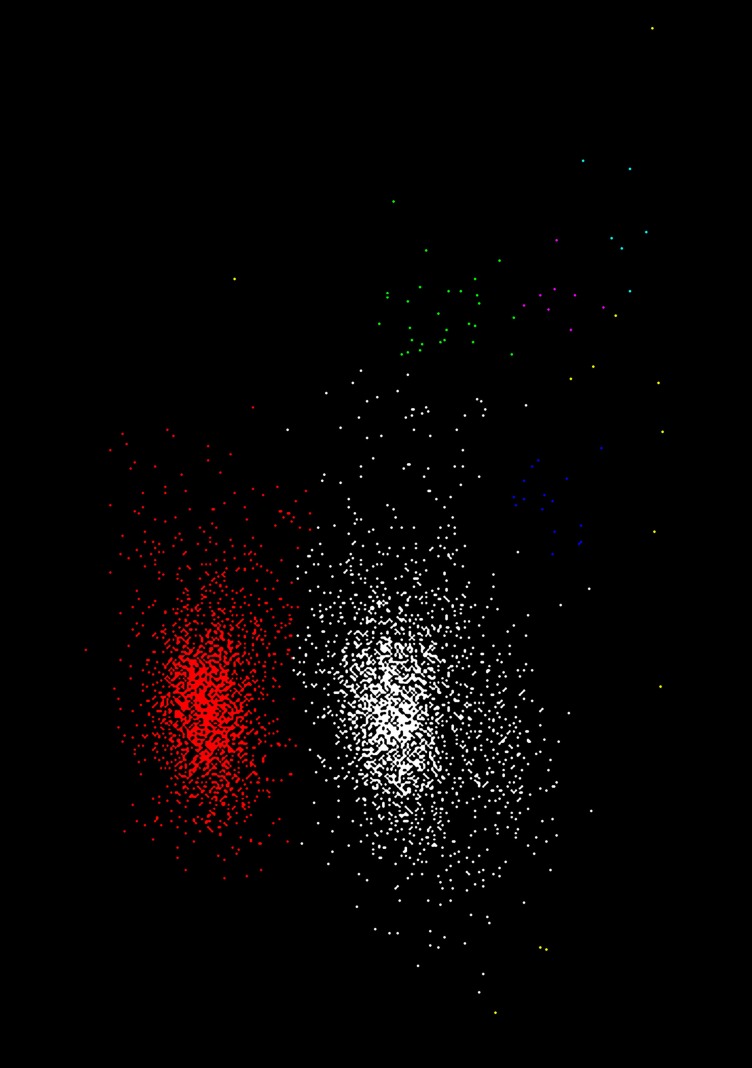
**The data points used in Table 2 with clusters colored according to the assignment in step 12**. The cluster with the highest stability score (*S*^1^ = 25) is shown in white; the second highest, cluster *C*^2^ (*S*^2^ = 21) is shown in red; the remaining clusters (green, dark blue, purple, cyan and yellow) are all less than threshold size and were later deleted. In this, and subsequent figures (Figures 5, 6, 8, 10, and 11) the *x* and *y* axes shows the first and second principal component values, respectively. Each point is a single event.

Table 2 shows the number of sub-clusters, *K*, their sizes, *C*^*k*^_*m*_, and stability scores, *S*^*k*^_*m*_, for a single clustering pass with varying values of σ_*m*_. As described above in section GAC Clustering Based on Principal Components this consists of a series of applications of the clustering algorithm with gradually increasing widths, σ_*m*_, of the Gaussian kernel gradient estimator (Equation 7). As σ_*m*_ increases in size, the number of sub-clusters decreases until only one is present. Ranges of σ_*m*_ within which sub-clusters remain stable in size and position were used to identify the presence of sub-clusters among the events. If no scores exceeded a threshold θ_*c*_, the cluster was deemed to be unsplittable. For cases where scores exceed threshold, one or more stable sub-clusters may be present and different choices of σ_*m*_ are possible. We normally chose the single most stable sub-cluster and split that off before repeating the pass on the remaining points. Figure 4 shows the points from which the values shown in Table 2 were obtained.

**Table 2:** **Values of clustering parameters obtained on a single pass of the GAC algorithm**.

***m***	**σ_*m*_**	***K***	**C^1^_m_[S^1^_m_]**	**C^2^_m_[S^2^_m_]**	**C^3^_m_[S^3^_m_]**	**C^4^_m_[S^4^_m_]**	**C^5^_m_**[*S***^5^_m_]**	**C^6^_m_[S^6^_m_]**	**C^7^_m_**	**C^8^_m_**
1	5	5	2496 [25]	1293 [4]	1244 [4]	71 [2]	51 [1]	44	39	37
2	5.5	6	2541 [25]	1286 [4]	1252 [4]	71 [2]	58 [1]	51 [1]	48	39
3	6.1	6	2664 [25]	1301 [4]	1276 [4]	84 [2]	63 [1]	55 [2]	32	32
4	6.7	5	2784 [25]	1318 [4]	1318 [4]	80 [2]	55 [2]	32	32	30
5	7.3	3	2908 [25]	2649 [21]	56 [1]	33	32	31	29	26
6	8.1	2	3036 [25]	2708 [21]	29	26	23	18	12	11
7	8.9	2	3037 [25]	2703 [21]	30	29	28	15	15	14
·	·	·	·	·	·	·	·	·	·	·
·	·	·	·	·	·	·	·	·	·	·
·	·	·	·	·	·	·	·	·	·	·
12	14.3	2	3153 [25]	2782 [21]	28	16	8	6	3	2
·	·	·	·	·	·	·	·	·	·	·
·	·	·	·	·	·	·	·	·	·	·
·	·	·	·	·	·	·	·	·	·	·
20	30.6	2	3182 [25]	2782 [21]	40	0	0	0	0	0
21	33.6	2	3223 [25]	2781 [21]	0	0	0	0	0	0
22	37	2	3223 [25]	2781 [21]	0	0	0	0	0	0
23	40.7	2	3222 [25]	2782 [21]	0	0	0	0	0	0
24	44.8	2	3222 [25]	2782 [21]	0	0	0	0	0	0
25	49.2	2	3226 [25]	2778 [21]	0	0	0	0	0	0
26	54.2	1	6004 [1]	0	0	0	0	0	0	0

*The value of σ_*m*_ increases by 10% at each step. The number of points in each of the clusters obtained for a given value of σ_*m*_ is shown, followed by the cluster stability score, S, in square brackets. Figure 4 shows the clusters present at step 12 (σ_*m*_ = 14.3)*.

**Figure 5:**
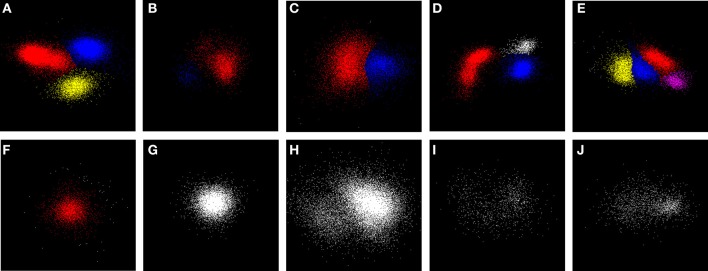
**Examples of clustering**. Panels **(A–E)** show examples of sub-clusters with high stability scores (*S* > 8). **(F)** shows an example of a single stable sub-cluster (red) surrounded by smaller clusters (white) which were less than the minimum cluster size, and were later deleted. **(G–J)** show stable clusters. **(G)** is an example of the final state of many clusters; **(H)** had a potential sub-cluster with a score *S* = 7 (points in the lower left-hand corner) that fell just below the clustering threshold of 8; **(I,J)** are irregular distributions whose scores also fell below threshold. Examination of the events in the irregular cluster (red) in **(D)** suggested that they came from a single unit whose height and shape varied over the period of recording. Sub-clusters were assigned with a choice of σ_*m*_ that lay in the middle of the range of values across which the number of stable sub-clusters was a maximum. These are ranked by size with colors in the order red, blue, yellow, and purple. Actual scores (in the same order) for each of the examples are: **A** (18, 25, 14), **B** (16, 12), **C** (14, 13), **D** (20, 15, 6), **E** (14, 14, 12, 8), **F** (15), **G** (7), **H** (7), **I** (3), and **J** (7).

Figure 5 shows examples of clustering outcomes for the GAC algorithm, including sets of points that were deemed to be unclusterable. Note the clustering of non-Gaussian distributions of points (e.g. the red points in Figure 5D) and that points in the tails or skirts of clusters are often included in the cluster (e.g. Figure 5B). While tails and skirts are often preserved, isolated, scattered points usually get deleted (Figure 5F). Boundaries between overlapping clusters are generally in the right places (e.g. Figure 5E) and, with the parameters chosen, irregular or sparse, non-concentrated distributions of points (Figures 5I,J) are usually not split.

**Figure 6:**
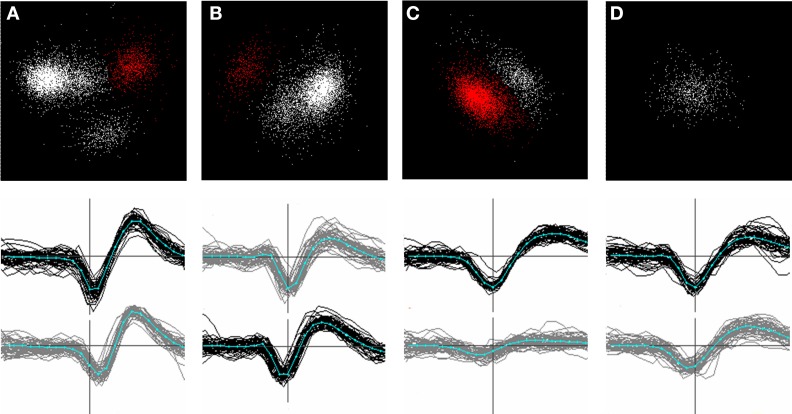
**(A–D)**: successive stages in splitting a channel-based cluster. Top row: clusters in principal components space. Bottom row: waveforms of the clusters shown in red in the top panel. Waveforms on the center channel are shown in black; waveforms on a neighboring channel in gray. The center channel in **(B)** has shifted because the spatial center of the template formed from these waveforms was closest to the lower channel. **(A)**: (top panel) the initial set of points (*n* = 6116) showing the single most stable cluster identified (red). After the points in this cluster are split off, a new principal components space is calculated from the remaining points **(B)** and the most stable cluster in this set is identified. This procedure is repeated until only one stable cluster remains **(D)**. Newly split clusters are subjected to the same procedure until all the clusters formed are judged to be stable. Note that the removal of a cluster in **(B)** results in clusters in the remaining points becoming more distinct **(C)**. Note that clustering is based on a larger number of channels than shown in the figure.

Whenever a new cluster was formed, or events removed from an existing one, a new principal components space was defined based on the remaining points in the cluster, and the clustering process was repeated. Figure 6 shows an example of a series of splits and re-projections of this nature. We frequently observed that removal of a single sub-cluster revealed new or more distinct clusters in the remaining points (e.g. compare Figures 6B,C).

### Merging and splitting clusters

**Figure 7:**
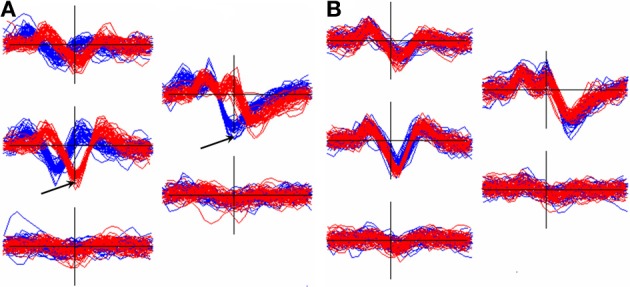
**Effect of alignment choice on spikes from a unit whose spike height was similar on two adjacent channels**. As a result, spikes were inconsistently registered to different channels and ended up in different clusters (superimposed waveform plots in red and blue). **(A)**: the effect of aligning the two templates to the negative trough on the center channel for each. Arrows show the center channels for the red cluster (5528 events) and the blue cluster (11314 events). The RMS difference, *q*, between the templates = 15.9 μV. **(B)**: the two clusters after separately aligning each to the 2nd derivative weighted measure of the template center (Equation 6, section Template-Based Alignment). Now *q* = 3.5 μV allowing the clusters to be automatically merged.

Events from a unit that is located midway between channels are likely to be inconsistently assigned to different channels and hence will end up in different clusters that need to be recombined. Figure 7 shows an example of this occurrence. The figure also illustrates the importance of aligning templates with a method whose dependence on the non-linear selection of features such as peak positions, or the center channel of the cluster, is minimized.

**Figure 8:**
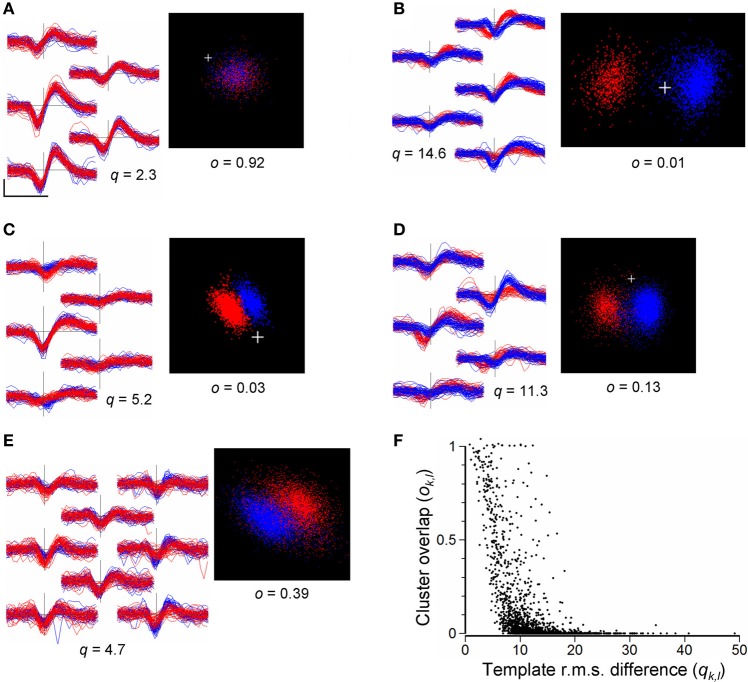
**(A–E)**: Comparisons of cluster pairs using the RMS waveform differences (*q*) and cPC overlap measure (*o*). Left panels show waveforms for the two clusters overplotted in red and blue with waveforms placed in positions corresponding to the recording sites. Right panels show the points in cPC space. **(A)**: example of a pair with similar waveform shapes and high cluster overlap, indicating that they should be merged. **(B)**: example of a pair with very distinct waveform shapes (*q* = 14.6) and low cluster overlap (*o* = 0.01) indicating that the pair is distinct. **(C)**: example of a pair with similar waveform shapes (*q* = 5.2) but distinct clusters (*o* = 0.03) in cPC space. **(D)**: a cluster pair with similar waveform shapes giving rise to two distinct but overlapping clusters in cPC space, indicating the need for merging followed by splitting. **(E)**: example of an ambiguous relation between two clusters: waveform shapes are similar and the combined set of points does not yield clearly separable clusters. Scale bar in **(A)** shows 0.5 ms and 100 μV, and applies to all waveforms. **(F)** Values of the cluster-pair overlap index *o*_*k*, *l*_ plotted against values of template RMS difference *q*_*k*, *l*_. Data are taken from a total of 1902 randomly chosen cluster pairs out of a total of 9510 in the 8 recordings shown in Table 3, immediately following the completion of splitting of channel-based clusters, and prior to automated or user-guided merging of clusters. The plus sign marks the origin in PC space.

As a guide to merging, we compared pairs of clusters, *k* and *l*, calculating the RMS difference, *q*_*k*,*l*_, between the waveforms (Equation 8) and the overlap measure, *o*_*k*,*l*_, (Equation 9) obtained from the distributions of points in the cPC space formed by temporarily combining the events in the pair. This was done only for cluster pairs with non-overlapping templates (see above in section Merging and Reassignment of Events Between Clusters). Figure 8F shows the distribution of values of *q*_*k*,*l*_ and *o*_*k*,*l*_ obtained for a total of 1902 such cluster pairs in the 8 recordings listed in Table 3. Overlap indices tend to be high (*o*_*k*,*l*_ > 0.5) for very small RMS differences (*q*_*k*,*l*_ < 5 μV), and small (*o*_*k*,*l*_ < 0.05) for large RMS differences (*q*_*k*,*l*_ > 20 μV). However, overall, values of *q* are not reliably predictive of *o* and vice versa. Cluster pairs with RMS differences <10 μV, indicating similarly shaped templates, could have high or low overlap indices (Figure 8). Although most pairs with large RMS differences (>15 μV) had low overlap indices (Figure 8B) some pairs could have high overlap indices (Figure 8F, points on the upper right of the distribution) suggesting that they might not be distinct in spite of their waveform shape difference. These observations indicate that the template RMS difference is an unreliable guide for deciding whether or not to merge clusters.

**Table 3:** **Summary of sorting results from 8 recordings made from 8 penetrations in 4 animals**.

**Recording ID**	**Stimulus**	**Recording duration (min)**	**No of events**	**Automated sorting time (min)**	**Initial no of distinct/total**	**Pairs to examine**	**Final no of distinct/total**	**% events classified**
17–32	S	45	3.3 × 10^5^	23	36/100	62	77/79	97.4
17–48	M	44	3.6 × 10^5^	13	8/41	37	30/30	98.0
18–09	S	17	1.7 × 10^5^	9	34/72	48	59/59	97.6
18–50	M	44	3.1 × 10^5^	47	26/76	58	51/55	95.6
21–03	M	44	1.3 × 10^5^	7	8/37	36	26/30	97.2
21–57	M	44	1.1 × 10^5^	6	18/60	44	46/48	96.1
22–17	M	44	2.7 × 10^5^	9	29/70	33	53/57	98.3
22–26	M	44	1.6 × 10^5^	33	23/117	90	72/83	93.7

*The recordings were of spontaneous activity (stimulus = S) or responses to m-sequence stimuli (stimulus = M). The time taken for user-guided merging and splitting was <10 min in all cases. Note that the total number of clusters drops during the user-guided stage due to merging of cluster pairs. The number of distinct clusters rises as a result of the user indicating that some pairs are distinct or the effects of merging or splitting. Parameters for the sorting were the same for all the recordings. For event detection, θ_*e*_ = 5.0; σ_*x*_ = σ_*y*_ = 70 μm and σ_*t*_ = 0.44 ms. The clustering thresholds were θ_*N*_ = 5% and θ_*c*_ = 8. Other parameters were as defined in the text*.

### Automated sorting

Table 3 summarizes overall sorting results from a representative set of recordings, showing the number of distinct clusters following the initial automated sorting stage and the number following the user guided stage. The time required for both stages was less than the recording time in most cases, though the ratio can be expected to rise for recordings with higher overall spike rates and spike numbers than those tested here.

**Figure 9:**
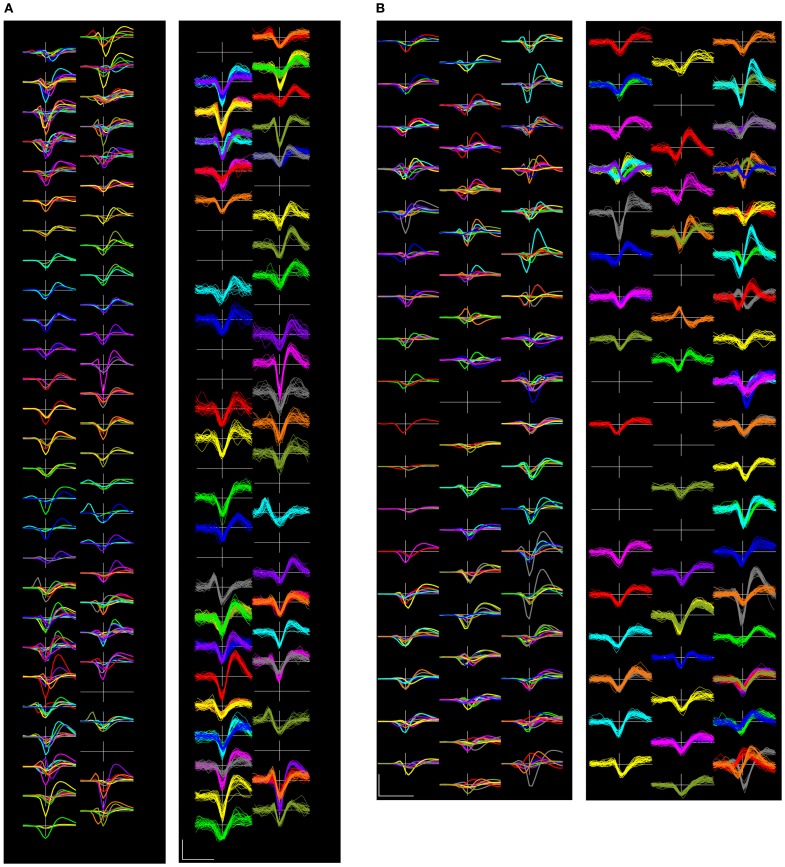
**Two complete sets of sorted template waveforms (left side of each pair) and superimposed individual event waveforms (right sides) from recordings (A): 18-09 and **(B)**: 22-27**. The template waveform displays show the templates for each of the units, including waveforms on the center channel and its immediate neighbors in colors that correspond with those used on the right panel. The event waveform displays show 25 randomly selected waveforms from each of the units on the center channel for each unit. Channels are laid out in their physical arrangement, though the horizontal spacing has been exaggerated for the purposes of display. Scalebar = 0.5 ms and 100 μV.

Figure 9 shows examples of complete sets of templates and randomly selected event waveforms present on the complete set of electrode channels in two representative recordings.

### Large amplitude spikes

**Figure 10:**
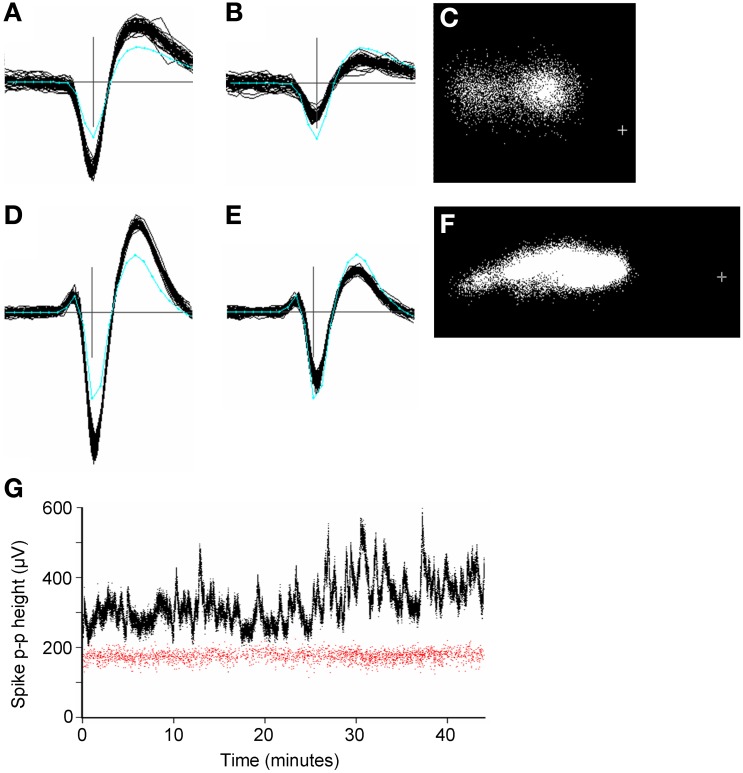
**(A–F)**: Spike waveforms from two units (panels **A–C** and **D–F**, respectively), whose height varied significantly over 45 min periods of recording. Panels **(A,B,D,E)** each show 50 superimposed waveforms, taken from periods when the height was a maximum **(A,D)** and minimum **(B,E)**. Only the center (maximum) channel of each unit is shown. The cyan line shows the average template waveform taken over the entire period of recording. The peak-to-peak amplitudes of the two templates were 205 and 325 μV. Panels **(C**, **F)** shows the distribution of the first two principal components of the spike waveforms. The cross marks the origin in PC space. In both cases the initial automated stage of clustering resulted in two clusters for the units which were merged in the user-guided stage. **(G)**: Plots of spike peak-to-peak height over time for two simultaneously recorded units (red and black dots). Each dot represents one spike. The variable unit (black dots) is the one shown in **(D–F)**. Note the lack of correlation in spike height variation for the two units. The two units were estimated to be 270 μm apart. The plus sign marks the origin in PC space.

Large amplitude spikes (e.g. > 200 μV peak-to-peak) could sometimes vary slowly and substantially in amplitude over the period of recording (Figure 10). Continuous variations led to clusters that were smeared out in PC space, often along the first principal component axis (Figures 10C,F). GAC could often tolerate such variations if they were gradual, however this was not always the case and spike height could sometimes vary discontinuously, resulting in the splitting of spikes into different clusters. These could normally be recombined in the user-guided stage based on the knowledge that the problem is common with large-amplitude spikes and the observation that the entire spike waveform shape changes in amplitude, rather than in any other shape aspect. Time course plots (Figure 10G) colored according to cluster origin, were also helpful in deciding that two units were actually more likely to be a single unit with a spike amplitude that was time-varying.

### Receptive field behavior

**Figure 11:**
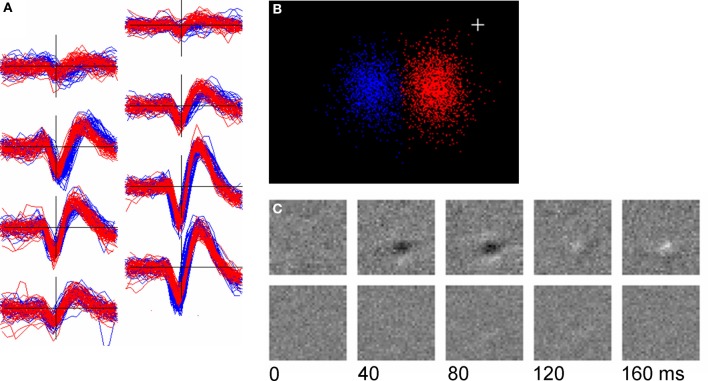
**Two units with similar spike shapes but different receptive fields. (A)**: overplots of 50 randomly chosen spikes from the two units (red and blue); **(B)**: Clustering of the two units in their cPC space; **(C)**: the receptive fields of the units determined by reverse correlation to an m-sequence stimulus. Top row is the red unit (2163 spikes) and the bottom row the blue unit (1318 spikes). The plus sign marks the origin in PC space.

Figure 11 shows an example (found with relatively little searching) of a pair of units whose template shapes were similar (*q* = 8.94) but which had clearly different receptive fields. Although spike shape was almost identical on the center channels (Figure 11A, bottom right), small differences in shape on the neighboring channels gave rise to two distinct clusters in cPC space (Figure 11B). One of the two units had a well-defined receptive field as determined by reverse-correlation to the m-sequence stimulus used during the period of recording (Figure 11C, upper row) while the other (presumably a complex cell) did not.

### Tests with surrogate data

Sorting was tested with surrogate data distributed to participants in a Workshop on Spike Sorting Software organized by G. Buzsaki and T. Harris, held at Janelia Farm Research Campus, USA, on February 24–26, 2013. Participants had no prior knowledge of the nature of the data or how results would be evaluated. Datasets were generated by taking recordings made with polytrode probes placed in the thalamus (8 site probe, staggered 20 μm spacing) or hippocampus (32 site probe, linear spacing) of freely moving rats (Peyrache et al., unpublished data). Spike signals for which there was “ground truth” were generated by taking well isolated spikes from a putative unit recorded on one shank and adding them to the recording on another shank thus, ensuring that the relationship of that spike train with background activity and brain states was preserved. All the recordings contained actual spiking activity in addition to the added ground truth spike trains. The quality of the spike sorting was judged by calculating the False Negative (FN) and False Positives (FP) for the ground truth spike trains. The FP rate for the 8 channel data (*n* = 6936 spikes) was 0.26% and for the 32 channel data (*n* = 7077 spikes) it was 0.014%. The corresponding FN rates were 2.1 and 0.37% (Peyrache, Personal Communication).

Additional tests were done on simulated ground truth data described in Quiroga et al. (2004) and available at http://www2.le.ac.uk/departments/engineering/research/bioengineering/neuroengineering-lab/software This data consists of a simulated single-channel recording with three different spike shapes added to noise backgrounds of variable amplitude. Classification results for these data for two different clustering methods, superparamagnetic clustering (SPC) and *K*-means, applied to the PCA distributions of the spike waveforms are given in Table 2 of Quiroga et al. (2004). We applied our event detection and clustering routines, without modification, to the simulated recordings after high-pass filtering with a cutoff at 0.5 KHz and a half-Gaussian roll-off with width σ = 0.25 KHz. The results are shown in Table 4 together with the values reported by Quiroga et al. (2004). They show that we detected and classified similar numbers of events as Quiroga et al. (2004) and that GAC made about a third (total = 4904) of the errors made by SPC (total = 16640). *K*-means clustering did better in some cases but we noted that the clusters in the simulated data (as shown by PCA) were approximately spherical, evenly distributed, and of almost equal size. These are ideal conditions for *K*-means but this algorithm would be expected to perform significantly less well with variably shaped and irregularly positioned clusters, as occurs in our non-simulated data. In addition, *K*-means, unlike GAC, is supervised, requiring seeding with the correct number of clusters. We also note that Quiroga et al. (2004) omitted event detection but instead used the known times of the spikes in the files. This means that FP are absent in their error rates. Our false positive rates were generally low, with the exception of files with noise levels of 0.2 or greater.

**Table 4:** **Comparison of different clustering algorithms applied to simulated single channel recording data (Quiroga et al., 2004)**.

**Data file**	**Noise**	**Classification errors**			**Number of spikes classified**		**Number of test spikes**
		**SPC**	***K*-means**	**GAC**	**QNB**	**SS**	
“Easy1_noise”	0.05	1	0	3 + 3	2729	2789	2797
	0.1	17	0	1 + 1	2753	2810	2831
	0.15	19	0	1 + 0	2693	2730	2760
	0.2	130	17	19 + 19	2678	2725	2756
	0.25	911	68	85 + 58	2586	2580	2645
	0.3	1913	220	193 + 147	2629	2379	2708
	0.35	1926^*^	515	388 + 473	2702	2028	2773
“Easy2_noise”	0.05	4	0	4 + 0	2619	2660	2668
	0.1	704	53	8 + 0	2694	2734	2747
	0.15	1732	336	162 + 10	2648	2679	2706
	0.2	1791^*^	740	926 + 186^**^	2715	2689	2775
“Difficult1_noise”	0.05	7	1	0 + 0	2616	2649	2659
	0.1	1781	184	77 + 0	2638	2715	2717
“Difficult2_noise”	0.05	1310	212	2 + 0	2535	2620	2637
	0.1	946^**^	579	104 + 0	2742	2793	2813
	0.15	1716^**^	746	891 + 15^**^	2631	2663	2695
	0.2	1732^*^	1004	963 + 165^**^	2716	2763	2763

The column headed “GAC” shows the number of classification errors, expressed as the total number of events in the final set of clusters that were from the wrong cluster, and the number of false-positives (i.e. events included in one of three clusters not present in the test set), for our combined event detection and clustering procedures. The two numbers are shown as a + b where a is the classification errors and b is the number of false positives. Asterisks show cases where only 1 (^*^) or 2 (^**^) clusters could be identified. High noise data files where both SPC and GAC failed to find more than one cluster are omitted. For comparison, errors reported by Quiroga et al. (2004) are shown for the same data sets clustered by K-means or SPC applied to the PCA distributions, together with the total number of spikes that was classified by Quiroga et al. (2004) (column “QNB”) and by us (column “SS”). The total number of simulated spikes in the datasets that we clustered, following removal of overlapping spikes as described in Quiroga et al. (2004) is shown in the column headed “Total test spikes.” The smaller numbers of spikes in column “SS” are due to events in the file that were not detected. Note that Quiroga et al. (2004) did not run an event detection stage but instead used the known times of the spikes in the files. For the sorting we used an event detection threshold θ_*e*_ = 2.8, with σ_*t*_ = 0.5 ms. Events were aligned to the positive peak. Clustering used the first 3 PCA dimensions, with a temporal window = ±0.75 ms around each spike. Clustering thresholds were θ_*N*_ = 5% and θ_*c*_ = 8. Other parameters were as defined in the text. Sorting times were typically less than a minute. In some cases a spurious fourth cluster consisting of false-positive events was manually removed, but this had no impact on the measurements in the Table.

## Discussion

It may not have been appreciated when polytrodes were first introduced (Drake et al., 1988; Bragin et al., 2000; Buzsáki, 2004; Blanche et al., 2005) that spike sorting might turn out to be a substantially more complex problem than with tetrodes. This has proved to be the case and can be attributed to the much higher dimensionality of the space in which spike signals must be detected and clustered as well as the greater volume of data that needs to be handled (Einevoll et al., 2012). An additional problem, not specific to polytrodes but also limiting, is the lack of a reliable, fast, algorithm for detecting clusters in the features extracted from the spike waveforms. The methods devised here address these and other difficulties specific to spike sorting with polytrodes: (1) alignment of events in such a way as to best reveal the presence of common features among them; (2) extraction of those low-dimensional features that are best diagnostic of differences between neurons; (3) clustering of these features to reveal putative single units; (4) post-clustering processing, including the need to merge clusters or reassign spikes between cluster pairs and finally (5) the need for an objective measure of how distinct each cluster is from all the others. Many of these problems also occur in the context of tetrode spike-sorting and multichannel electrodes in general. They are separable, which means that improvements in any one of them can be expected to lead to improvements in the overall procedure. We discuss our particular solutions of them in the same order, as follows.

### Alignment of events and templates

We found that accurate definition of event times, interpolating to within a few μs, was critical to clustering and avoiding artifacts. For example if interpolation was not done, large amplitude spikes were sometimes split into two spurious clusters (data not shown) if the peak was used for alignment. The choice of an alignment criterion was difficult because of the variability in event shapes, both within and between units. Alignment can be done in two ways: either with reference solely to the event waveform—event-based alignment—or with reference to a template which is the average of many similar waveforms. Event-based alignment has to be done immediately following event detection since reliable templates do not exist yet. Since it is based on information in only a single waveform, it is susceptible to noise, especially for events of low-amplitude. Before events can usefully be aligned with respect to a template there needs to be some certainty that the events from which the template is formed are already approximately well aligned and similar to each other. However, this condition is generally met following the initial clustering step. We tested a variety of specific alignment criteria for event and template alignment, including alignment to the most negative trough, the most positive peak, the time of the positive going zero-crossing and the 2nd derivative weighted estimate used in Equation 6. As argued above, measures that depend on a non-linear selection of a feature such as a peak or a zero-crossing have the disadvantage that a small change in waveform shape may result in a large change in alignment time. This can result in spurious clusters. Measures of mean position are not susceptible to such effects but they seemed susceptible to noise, especially for low-amplitude events, when used for event-based alignment. Hence we compromised, using alignment to the negative trough for initial event-based alignment, followed by least-squares matching to the template and then template-based alignment to mean position as soon as clusters were split off from the initial channel-based set by the GAC algorithm. Other initial alignment choices however did not give radically worse results.

### Extraction of low-dimensional features

Following event detection, clusters were initially defined as sets of events registered to a specific channel on the electrode. We then attempted to split each of these clusters. For this, and all subsequent clustering steps, we chose to use principal components analysis (PCA) to extract features to use in clustering, using interpolated voltage values from waveforms on the channels assigned to the template, taken within a time window of ~1 ms centered on each event. The first two or three principal components were generally found to be adequate for revealing clusters. Clustering time scales linearly with the number of feature dimensions so additional components might be used with relatively little penalty. However, we have, so far, no clearly documented evidence of an improvement in clustering produced by using more than three components. Other methods, such as wavelet analysis (Quiroga et al., 2004) or independent components analysis (ICA; Comon, 1994; Lewicki, 1998; Hyvärinen and Oja, 2000) might be used to extract features at this stage. While PCA is able to detect remarkably small differences in spike waveform shape (Figure 11) ICA (which is much more computationally intensive) seems better at separating clusters of very unequal size (Spacek, unpublished observations). Feature selection methods that make assumptions about the normal shapes of spikes (for example fitting a mathematical model of spike shape to each event and using the model parameters as features) face a problem in that we quite often found units (the one shown in Figure 7 is an example) with unconventional shapes—waveforms that are not similar to any of the shapes shown in Figure 3. It is unlikely that model based feature extraction, or possibly also wavelet analysis, would detect such shape variations. PCA or ICA analysis on the other hand embodies no assumptions about the shapes of the events to be classified.

### Variability in spike shape

Spike shape, especially amplitude, can change over time and can result in spurious clusters. The changes likely have varying causes. We sometimes observed erratic increases and decreases in peak-to-peak height in large amplitude spikes. The same variations were not observed in smaller simultaneously recorded units (Figure 10). Perhaps neurons with larger amplitude spikes are physically closer to the electrode than other units and are thus more susceptible to movement and other artifacts; otherwise it is difficult to ascribe a cause to the variations. We sometimes observed slow changes in waveform shape that were correlated with shifts in the estimated positions of many different units (section Calculation of Templates); these were most likely due to accidental shifts in electrode position (Spacek and Swindale, unpublished data). Occasionally, a small cluster of spikes differed from a larger cluster only by discrete variation in a small portion of the waveform on a single channel, suggestive of all-or-none dendritic spikes (Buzsáki and Kandel, 1998). Variations observed by others include decreases in spike amplitude during bursts or over short (<50 ms) inter-spike intervals (Fee et al., 1996b; Harris et al., 2000; Henze et al., 2000). Systematic slow decreases in height over longer periods of time, sometimes over periods of minutes of prolonged firing have been observed in hippocampal recordings (Quirk and Wilson, 1999). Such systematic effects on spike height did not seem common in our visual cortex recordings, possibly because the neurons tended not to fire in bursts or at prolonged high rates. GAC is relatively insensitive to such variations, especially if they result in smooth elongations of cluster shape (Figure 10F). However, height and shape variations can occasionally be discontinuous and the resulting clusters can only be recognized as likely to belong to the same unit by the user—and even then not always with complete certainty. One way of mitigating the effect of variations in large amplitude spikes might be to rescale voltage values using a compressive transform. Another is to use the normalized dot product (or correlation) as a way of detecting similar template pairs since this measure is insensitive to multiplicative changes in voltage scale. Severe shape variations causing waveform shapes of different units to overlap (so that the shape of one at the start of a recording period might resemble the shape of another at the end) might be dealt with by including event time, appropriately scaled, as one of the feature dimensions used in clustering. The logic of this is that such clusters may be separable at each point in time, but not if points are collapsed across the time axis. Other methods for clustering that take response variability into account, but which we did not attempt to implement, have been proposed (Pouzat et al., 2004; Bar-Hillel et al., 2006; Calabrese and Paninski, 2011).

### Gradient-ascent clustering

The GAC algorithm we have used here appears to have significant advantages over existing clustering methods and has not, to our knowledge, previously been applied to spike sorting in the form used here. Conceptually, GAC defines a cluster as a collection of points distributed around a central location, with a density that falls off monotonically along trajectories leading outwards from the center. Boundaries between clusters are density minima or saddle points where the direction vector (Equation 7) has zero length. The density gradient is estimated with a Gaussian kernel with a width σ_*m*_. The width is chosen so that the positions and sizes of the clusters that are found remain within defined limits across a range of values of σ_*m*_. This may be regarded as a form of scale-invariance, a property that has been found useful in identifying visual features in images (Lowe, 2004). In addition to satisfying an intuitive definition of a cluster, the GAC method has a number of advantages. Unlike *k*-means, the number of clusters does not need to be specified in advance, and boundaries between clusters are not necessarily drawn halfway between lines joining their centers. Unlike algorithms based on Gaussian mixture models (Harris et al., 2000; Litke et al., 2004) no restrictions are put on the shapes of clusters. Although clusters in our data were often Gaussian in shape, this was not always the case. Height variations in particular tended to produce non-Gaussian distributions and other clusters often had tails or skirts which it seemed desirable to include. The GAC algorithm is also quite fast. As shown in Table 3, we were able to cluster recordings with several hundred thousand spikes typically in 30 min or less. Because each scout point has to sum over all the other points in the sample (Equation 7) GAC execution time should scale as the square of the number of sample points. We avoided this by summing over a subset of the points (section GAC Clustering Based on Principal Components). Additional ways of speeding up the code can be envisaged. For clustering in two dimensions, data points can be represented not as a list of position values but as a density distribution in a two-dimensional array. Depending on the size of the array there will be some loss of resolution of position values but this seems unlikely to have a substantial effect on the accuracy of placement of cluster boundaries.

Although the GAC algorithm was independently developed by us, many clustering algorithms use related approaches. An early approach used a technique known as gravitational clustering (Butler, 1969; Wright, 1977) in which points aggregate under mutual attraction. The procedure of using a Gaussian kernel density estimator to move up a density gradient of fixed data points was first devised by Fukunaga and Hostetler (1975) and is elsewhere termed the mean-shift algorithm (http://en.wikipedia.org/wiki/Mean-shift). This algorithm was recently applied to spike sorting of single channel data by Zhao et al. (2010) and to multi-electrode recordings from the retina by Marre et al. (2012). Other similar gradient-based methods have been proposed for clustering problems, e.g. by Kowalewski (1995) and Wang et al. (2004). However, the present paper adds several features to the mean-shift algorithm that make it very much more tractable for spike sorting. Merging scout points following each position update speeds up the algorithm substantially (the exact amount depends on a variety of factors but can be two orders of magnitude for >20,000 data points). Our procedure for automatically choosing the bandwidth parameter σ_*m*_ (section GAC Clustering Based on Principal Components) and the assignment of a stability score to different clusters removes the need for the user to choose a suitable value, while at the same time it allows a degree of flexibility in the clustering strategy. For example it allows for the strategy used here of defining and splitting off the single most stable (distinct) cluster and then re-clustering the remaining points. To the best of our knowledge these particular strategies have not been used before in the context of spike sorting.

As well as comparing SPC (Blatt et al., 1996; Quiroga et al., 2004) with GAC on surrogate data (section Tests with Surrogate Data, Table 4) we tested it on our own data but found that it frequently was unable to separate distributions that were connected by narrow bridges of low density. Part of the appeal of this algorithm is that it is extremely insensitive to density variations and cluster shape. However, this behavior seemed unsuitable for many of the types of cluster that we encountered in our data.

We have not attempted a full comparison of GAC with the many other clustering methods that have been proposed (e.g. see http://scikit-learn.org/dev/modules/clustering.html). As clustering is only one step in the processing chain proposed here (albeit an important one) it might, in principle, be replaced by some other method.

### The merging problem

Recognition of template/cluster similarity for the purposes of merging turned out to be quite difficult. We found that the RMS difference between template waveforms (*q*_*k*,*l*_, Equation 8) was not an adequate measure to use on its own for merging clusters. We devised a different measure of cluster similarity by projecting the data points in the two clusters into the cPC space formed by combining the two clusters and making a quantitative measure of the degree of overlap of the two distributions (*o*_*k*,*l*_, Equation 9). If *q*_*k*,*l*_ is an adequate measure of template similarity it should reliably predict *o*_*k*,*l*_, however it does this only weakly (Figure 8F). Within the major part of the range of values of *q*—between about 5 and 20 μV—values of *o*_*k*,*l*_ were broadly distributed, with values <0.1 indicating cluster pairs that were extremely distinct and values >0.9 indicating that clusters pairs were almost certainly not distinct and should be merged. Extremely small values of *q* (<5 μV) and very large values (>25 μV) could safely be used as a criterion, but this accounts for only a small number of the possible pairs. Hence we relied largely on the cPC overlap value as a basis for merging and for declaring cluster pairs to be distinct. RMS differences are widely used as a way of matching spikes to templates (e.g. Segev et al., 2004) however, our analysis suggests that the procedure will sometimes fail to match spikes to a template to which they belong (i.e. large values of *q* can accompany large values of *o*) while spikes belonging to different units may be wrongly matched to the same template (low values of *q* accompanying low values of *o*). There are two possible reasons for insensitivity of the RMS measure. Firstly, RMS measures the distance between the means of the waveforms (or between a single waveform and the mean of another set) and does not take the distributions of the waveforms, or their correlations, into account. Secondly, RMS measures distance in the voltage space of the two waveforms whereas the overlap measure is based on distances measured in only the first two principal components dimensions, which ignores much of the variance, presumably increasingly due to noise, in the additional higher dimensions. Hence the RMS measure may simply be too diluted by noise to be useful for assessing whether spikes, or templates, derive from the same unit.

### Cluster distinctness

We were able to extract clusters from our data which we can say are objectively unitary entities inasmuch as they are separable by our clustering algorithm and pass test *DT*, (section Pairwise Distinctness Tests) for distinctness from all other clusters in the recording. Some qualifications need to be attached to this however. Spikes that end up in clusters less than the threshold size may be deleted before merging and hence will be lost. This problem can be minimized by choosing a smaller minimum cluster size at the expense of having to deal with more small clusters. A second problem is that the GAC algorithm is capable of separating clusters whose distributions overlap at the edges. Even though this means the pair is defined as distinct, some spikes in each will be in the wrong cluster. It is difficult to be sure how many without making assumptions about cluster distributions which may not always be correct. Even when this problem does not occur, we have no objective way of knowing that clusters represent all, or the majority, of the spikes from only a single neuron. An irregular, or a non-Gaussian distribution of principal components or other feature value does not necessarily mean that a cluster is multi-unit. This is demonstrably the case for many large amplitude spikes, but might be true for smaller amplitude spikes as well. Such irregular, non-Gaussian distributions imply that, for our data, the quality metrics for spike sorting proposed by Hill et al. (2011) would be of limited use. Conversely, a homogenous Gaussian distribution may suggest that only a single unit is present but one cannot be sure of this. The differences in spike shape between different neurons can in some cases be small (Figure 11) and might in other cases be even smaller. Nor can one be certain that different clusters are necessarily always different units. The spikes produced by a single neuron might vary in shape, possibly bimodally, resulting in spikes from a single unit being wrongly split into two clusters. We did sometimes observe cluster pairs, one typically with many more events than the other, which were distinguished only by variations in the spike waveform preceding the initial phase of the spike. We generally decided to merge such pairs. It is conceivable that spike shapes might differ depending on the way in which the spike was initiated within the cell (for example whether stimulus driven or spontaneously generated), or depending on the presence of a backpropagating action potential. Although perhaps unlikely, this is a possible explanation of the results in Figure 11. It is also possible that backpropagating (Buzsáki and Kandel, 1998) or other dendritic spikes or other local non-linear phenomena might variably change spike shape.

Ultimately, the validity of this and other spike sorting methods, and arguably of all types of extracellular recording that claim to isolate single neurons, depends on the presumption that waveform variations due to the smallest position differences between pairs of cells are reliably larger than, or detectably different in kind from, intrinsic variations within cells over time. This presumption will be hard to verify. The relevant position differences will often be small (e.g. 10–20 μm) since neuronal cell bodies in the cortex can abut (Feldman, 1984). Turning the problem on its head, Mechler et al. (2011) estimate that, with tetrodes, the voltage signal from a single neuron allow it to be localized within a sphere of ~50 μm radius. This has the corollary that it may be hard or impossible to separate signals from neurons that are less than 50 μm apart. Tests in which a single neuron is recorded both intra- and extra-cellularly (Harris et al., 2000), though they yield valuable data about the completeness of sorted clusters, do not seem adequate to answer this question. Simultaneous intra- and extra-cellular recordings from pairs of neighboring neurons, perhaps offer the best chance of answering the question but may be very hard to do, especially *in vivo*.

### Overlapping spikes

Our procedure does not attempt to identify or sort temporally overlapping spikes. Hence spikes from units that are physically close (e.g. <100 μm apart) and that occur within 1 ms of each other (which is roughly the temporal resolution of our event detection method) are unlikely to be sorted correctly. Unless the temporal firing relationship of the pair is determinate to within fractions of a millisecond (which seems very unlikely) the superimposed waveforms will be variable in shape and are most likely to end up as outlying points in the cluster distributions and will be deleted. Such overlapping pairs are unlikely to be a large fraction of the spikes for any given unit. For example, for a pair of units firing at a relatively high rate (for visual cortex) of 20 Hz only about 2% of the spikes in each of the pairs would be expected to overlap and be lost. In addition to this, for measures of correlation between cell pairs that used a binwidth of the order of 10 ms, only about 10% of the spikes in the most central (Δ*t* = ± 5 ms) bin would be expected to be lost. Hence, even for measures of cross-correlation, the consequences of losing overlapping spikes may often not be severe. The limitation does not apply at all to pairs of units that are >100 μm apart, and this constitutes the majority of cell pairs in any given recording. However, methods for resolving overlapping spikes have been proposed (Lewicki, 1994; Segev et al., 2004; Franke et al., 2010; Marre et al., 2012) and these might perhaps be applied to the classification of events rejected by our sorting procedure (typically around 5–10% of the total: Table 3).

### Application to tetrodes and MEAs

Although developed as a method for sorting spikes recorded with 54 channel polytrodes, our methods are applicable to spike sorting with other kinds of multichannel electrode, including tetrodes. An approach to sorting single or multiple tetrode recordings, which we have explored in preliminary tests, is to form 4 clusters per tetrode initially, assigning events to the tetrode site on which the event peak-to-peak amplitude is greatest. Each set of four clusters is then split and recombined in the same way as done for polytrodes following the initial formation of channel-based clusters (section Formation of Channel-Based Clusters). Because our method uses the “divide and conquer” approach of defining clusters based only on the voltage waveforms present on a center and a limited number of nearby channels, the time taken for the initial splitting of channel-based clusters should scale linearly with the number of channels. The second stage of sorting, which is based on pairwise tests, should also scale linearly because although the number of pairs increases with the square of the number of channels, only those pairs that are located close to each other enter directly into the comparisons (rule 1 of test *DT*, section Pairwise Distinctness Tests). Hence processing time for the second sorting stage should also scale linearly with the number of sites. A potential limitation to the method exists in the case of arrays with very closely spaced sites. Here it is possible that signals from single neurons might be variably assigned to several adjacent sites (with the design of polytrodes that we have tested it is rarely more than two) resulting in excessive splitting of single units into multiple clusters. Recombining these one pair at a time might prove to be time-consuming, especially if done during the user-guided stage. Possible alternative strategies might be to assign events to virtual sites, more coarsely spaced than the real ones, or to exploit the greater spatial resolution of closely spaced arrays and form initial clusters based on estimates of the position of each event in the *x-y* plane of the electrode.

### Conflict of interest statement

The authors declare that the research was conducted in the absence of any commercial or financial relationships that could be construed as a potential conflict of interest.
